# Frontal fibrosing alopecia coexisting with vitiligo: is there a true association?^☆^

**DOI:** 10.1016/j.abd.2021.10.018

**Published:** 2023-07-19

**Authors:** Jéssica Pauli Damke, Bruna Ossanai Schoenardie, Rochelle Figini Maciel, Juliano Peruzzo

**Affiliations:** Serviço de Dermatologia, Hospital de Clínicas de Porto Alegre, Porto Alegre, RS, Brazil

*Dear Editor,*

The coexistence of vitiligo and Frontal Fibrosing Alopecia (FFA) has already been reported,^1^, ^2^ however, it is still uncertain if there is a true association between both conditions.

A 58-year-old woman was referred to treat vitiligo, which she had for 6 years. Upon examination, we noticed hair rarefaction on the frontal region of the scalp, on the same topography of achromic patches of vitiligo (Fig. 1A). A scalp biopsy showed interface dermatitis restricted to the pilous infundibulum with numerous apoptotic cells and incipient perifollicular fibrosis, which confirmed the FFA diagnosis. Both conditions manifested after menopause. On follow-up, eyebrows rarefaction was noted concomitant with a growing vitiligo patch (Fig. 1B).

**Figure 1:**
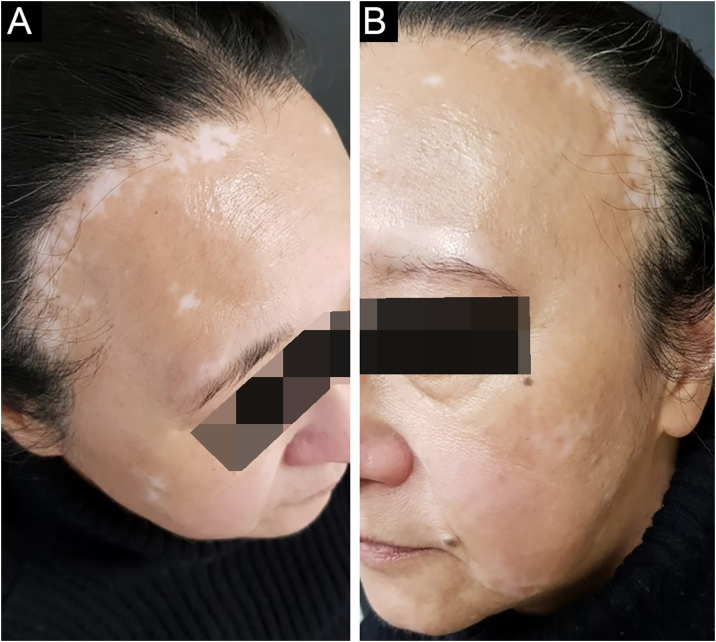
Clinical findings of patient 1. (A) Hair rarefaction on the frontal region of the scalp, coexisting with achromic macules of vitiligo. (B) Eyebrows rarefaction on a growing vitiligo macule

A 75-year-old woman, who had a diagnosis of vitiligo since she was 40 years old, presented with complete depigmentation of the skin. She also had frontal alopecia with an atrophic aspect of the scalp, a pseudo-fringe sign and almost complete loss of hairs in both eyebrows (Fig. 2).

**Figure 2:**
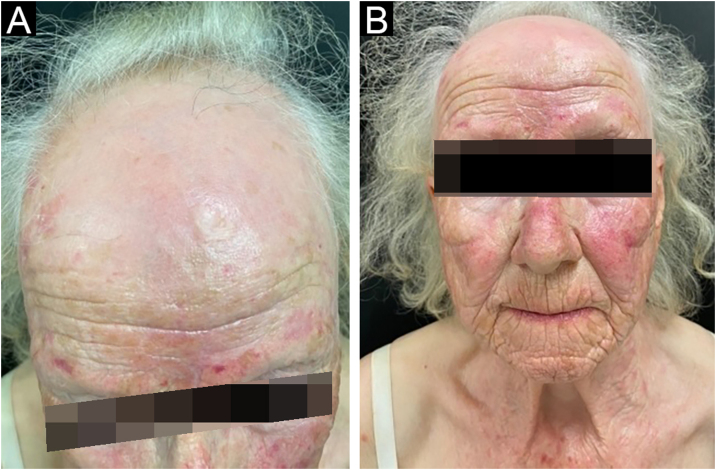
Clinical findings of patient 2. (A-B) Complete depigmentation of the skin, accompanied by frontal alopecia with an atrophic aspect of the scalp, pseudo-fringe sign and almost complete loss of hairs in both her eyebrows

FFA is a chronic lymphocytic cicatricial alopecia that characteristically affects the frontotemporal hairline and frequently also the eyebrows. It is seen predominantly in postmenopausal women, and it is more common in Caucasian patients. Some authors consider FFA a possible clinical variant of Lichen Planus (LP),^3^ due to the similarity of histopathological findings. Since FFA progresses very slowly, it is often difficult to precisely define when it first began.^4^

Vitiligo is an autoimmune disease characterized by a selective loss of melanocytes, which causes cutaneous depigmentation. It is clinically characterized by achromic macules. Genetic and environmental factors are involved in its development.^5^ The association between them has already been described.^1^, ^2^ More recently, in a cohort of 20 patients diagnosed with FFA, two of them presented also with vitiligo.^1^

Vitiligo has been associated with LP, which might be explained by the finding of a CD8+ cytotoxic inflammatory infiltrate in both conditions.^1^ The association between FFA and vitiligo might lie in the fact that FFA could be a variant of lichen planopilaris.^3^ It is known that melanocytes and keratinocytes form functional units. So it has been postulated that since keratinocytes in the outer root sheath are continuous with epidermal keratinocytes, they probably express the same adhesion molecules in which the lymphocytes attach,^2^ leading to a lymphocytic attack on the melanocyte-keratinocyte units and explaining the physiopathology of both diseases. Additionally, both vitiligo and LP are known to present the Köbner phenomenon, which might explain the presence of both in the same topography.^1^

The concomitant onset of a vitiligo patch and FFA on the eyebrow of our first patient reinforces that there might be an association between them. Furthermore, the second patient who had a more extensive case of vitiligo also had a more advanced case of FFA, which might strengthen the hypothesis that the pathophysiology of both conditions may be linked. However, more studies are required to elucidate the exact mechanisms through which these two relate.

## Financial support

None declared.

## Authors' contributions

Jéssica Pauli Damke: The study concept and design; data collection, or analysis and interpretation of data; writing of the manuscript or critical review of important intellectual content; data collection, analysis and interpretation; intellectual participation in the propaedeutic and/or therapeutic conduct of the studied cases; critical review of the literature; final approval of the final version of the manuscript.

Bruna Ossanai Schoenardie: The study concept and design; data collection, or analysis and interpretation of data; writing of the manuscript or critical review of important intellectual content; data collection, analysis, and interpretation; intellectual participation in the propaedeutic and/or therapeutic conduct of the studied cases; critical review of the literature; final approval of the final version of the manuscript.

Rochelle Figini Maciel: The study concept and design; data collection, or analysis and interpretation of data; writing of the manuscript or critical review of important intellectual content; data collection, analysis and interpretation; intellectual participation in the propaedeutic and/or therapeutic conduct of the studied cases; critical review of the literature; final approval of the final version of the manuscript.

Juliano Peruzzo: The study concept and design; data collection, or analysis and interpretation of data; writing of the manuscript or critical review of important intellectual content; data collection, analysis and interpretation; effective participation in the research guidance; intellectual participation in the propaedeutic and/or therapeutic conduct of the studied cases; critical review of the literature; final approval of the final version of the manuscript.

## Conflicts of interest

None declared.

## References

[1] Katoulis A.C., Diamanti K., Sgouros D., Liakou A.I., Alevizou A., Bozi E. (2017). Frontal fibrosing alopecia and vitiligo: coexistence or true association?. Skin Appendage Disord.

[2] Miteva M., Aber C., Torres F., Tosti A. (2011). Frontal fibrosing alopecia occurring on scalp vitiligo: report of four cases. Br J Dermatol.

[3] Poblet E., Jiménez F., Pascual A., Piqué E. (2006). Frontal fibrosing alopecia versus lichen planopilaris: a clinicopathological study. Int J Dermatol.

[4] Lis-Święty A., Brzezińska-Wcisło L. (2020). Frontal fibrosing alopecia: a disease that remains enigmatic. Postepy Dermatol Alergol.

[5] Bergqvist C., Vitiligo Ezzedine K. (2021). a focus on pathogenesis and its therapeutic implications. J Dermatol.

